# Prevalence of trachomatous inflammation-follicular and associated factors among children aged 1-9 years in northeastern Ethiopia

**DOI:** 10.1186/s12887-024-04587-4

**Published:** 2024-02-19

**Authors:** Tadesse Altaseb, Mistir Lingerew, Metadel Adane

**Affiliations:** 1 Jamma District Health Office, South Wollo Zone, Amhara Region, Ethiopia; 2 Department of Environmental Health, College of Medicine and Health Sciences, Wollo University, Dessie, Ethiopia

**Keywords:** Prevalence, Trachomatous inflammation-follicular, Rural children aged 1–9 years, Jamma district, Ethiopia

## Abstract

**Background:**

Trachoma is the most prevalent eye disease in Ethiopia, especially among children aged 1–9 years and continues to be a public health concern. Nevertheless, in Ethiopia’s rural Jamma district in South Wollo Zone of Amhara Regional State, factors associated with trachomatous inflammation-follicular (TF) among children aged 1-9 years have not yet been studied.

**Methods:**

A community-based cross-sectional study was conducted among 616 children aged 1–9 years in rural Jamma district in Ethiopia from January-March, 2019. Data were collected using a pre-tested structured questionnaire, an observation checklist and clinical examination of study participants for active trachoma. The presence of TF and trachomatous inflammation-intense (TI) was clinically assessed by integrated eye care workers using the World Health Organization simplified grading system. Data were analysed using SPSS (Statistical Package for Social Sciences) Version 25.0. A logistic regression model with 95% CI was used. From the multivariable analysis, variables with *p*-value < 0.05 were declared as associated factors of TF.

**Result:**

The prevalence of TF was 10.9% (95% CI [8.6 - 13.6%]) among the rural children aged 1-9 years. The mean family size was 5.5 ± 1.9 persons. About one-fifth (20.6%) of households kept domestic animals overnight in the same room as family. Almost one-sixth (17.5%) of the children involved in this study had an ocular discharge. Two-thirds of the children (68.8%) washed their hands once per day and just over half (55.8%) washed their faces once per day. From multivariable analysis, we found that the presence of domestic animals kept overnight in the same room as the family (adjusted odds ratio [AOR] = 4.32; 95%CI [2.49–9.52]), mother’s/caregiver’s illiteracy (AOR = 2.01; 95%CI [1.11–4.67]), household size (> 7 persons) (AOR = 3.50; 95%CI [1.66–8.50]), washing of children’s hands and face without soap (AOR = 2.41; 95%CI [1.29–5.18]), feces observed in the compound (AOR = 5.10; 95%CI [2.01–10.14]), presence of ocular discharge (AOR = 7.23, 95%CI [4.10-12.51]) and nasal discharge (AOR = 4.54, 95%CI [1.95–9.26]) were significantly associated with TF.

**Conclusion:**

The prevalence of TF among rural children aged 1–9 years in this study was almost two times higher than the WHO-recommended threshold (TF < 5%) for trachoma elimination and beyond the trachoma control target (TF < 10%). Presence of domestic animals kept overnight in the same room as the family, mother’s/caregiver’s illiteracy, household size (> 7 persons), washing of children’s hands and face without soap, feces observed in the compound, presence of ocular and nasal discharge were significantly associated with TF. We recommended interventions that will help household income to be improved to enable families to be able to construct separate rooms in which to keep animals overnight. Furthermore, we also recommend to policy makers to design mechanisms for enhancement of behavioural change among householders to keep household compounds clean and creating awareness among mothers/caregivers about prevention of trachoma.

**Supplementary Information:**

The online version contains supplementary material available at 10.1186/s12887-024-04587-4.

## Background

Trachoma is an eye disease that causes characteristic inflammatory responses, leading to scarring of the inner surface of the eyelids that can lead to blindness if left untreated [1]. It is caused by repeated *Chlamydia trachomatis* infections [2]. Trachoma primarily affects the poorest and often isolated communities that lack access to clean water, sanitation and basic health services [3]. Routes of transmission include direct eye-to-eye spread (e.g. while playing together or sharing a bed), fingers, and indirect spread through sharing towels and pillows [4–7].

According to the World Health Organization (WHO), implementation of trachoma elimination activities are recommended based on SAFE strategies (Surgery, Antibiotics, Facial cleanliness, and Environmental improvement) in areas where trachomatous inflammation-follicular (TF) is 5% or above, and trachomatous trichiasis (TT) among adults ages 15 years or above is ≥ 0.2% [8]. Moreover, implementation of SAFE strategies are a higher priority in areas where TF ≥ 10% and (TT) ≥ 1% than those areas with lower rates that are still above elimination thresholds [8, 9]. Worldwide, approximately 200 million people live in areas where trachoma is now confirmed endemic [10]. In 2018, a total of 177.8 million people lived in the 1477 districts worldwide in which the TF prevalence in children aged 1–9 years was ≥ 5%. A total of 89.1 million people received antibiotics for elimination of trachoma in 2018, compared to 83.5 million people treated in 2017 [8].

The highest prevalence of active trachoma and trichiasis remains in the Sahel area of West Africa and Savannah areas of East and Central Africa [11]. In Ethiopia, trachoma is highly endemic [12], Although prevalence findings are lower than those found previously, recent studies in Ethiopia [13–15] show that prevalence is still above WHO’s standard of trachoma elimination goal of less than 5%, which indicates trachoma is still of public health interest in Ethiopia [16].

A trachoma impact survey in Amhara Region, Ethiopia from 2010 to 2015 found that the prevalence of TF and TI among children aged 1–9 years was 25.9% and 5.5%, respectively [17]. A study of the burden of and factors associated with active trachoma in the North and South Wollo Zones of Amhara Region found a high prevalence of 21.6%, a rate that is four times higher than the WHO elimination goal (TF prevalence < 5%) [13].

Although there have been several studies done in Ethiopia recently [13–15, 18–22], their findings varied due to differences in the socio-economic levels of their source populations, study areas (rural verses urban), the examined population (children verse adult or both) and differences in the methods of eye examination. Although elimination targets are considered at the district level (WHO definition of district is an administrative area of approximately 100,000-250,000 inhabitants) [4], gathering data at local levels is essential; given that trachoma prevalence in many areas is still quite high, there is a need for area-specific evidence to further strengthen trachoma elimination programs in the various regions of Ethiopia, in particular in rural South Wollo Zone.

For more than a decade, a trachoma control and elimination program has been implemented by the Amhara Regional Health Bureau, Carter Centre- Ethiopia, Organization for Rehabilitation and Development in Amhara and Christian Blind Mission (CBM) with support from several partners and donors [23]. Some of the trachoma projects that were implemented included Amhara Trachoma Control and Elimination Program, Amhara Trachoma Control Program, Amhara Trachoma Elimination Program, Amhara Neglected Tropical Disease Program. However, in many woredas (districts) of the region including rural Jamma, the prevalence of TF in children is still above the elimination target set by WHO [17] and the reasons for this are unknown.

Thus, to further effectively control and eliminate trachoma in the area, guidance may be provided by the first-hand evidence of this study. Our study will help to strengthen the current evidence or reinforce previously identified key findings from another district. Therefore, this study was conducted with the objective of determining the prevalence of TF and associated factors among Jamma’s rural children aged 1–9 years, which will help to guide intervention measures to tackle the problem.

## Method and materials

### Study area description

The study was conducted among rural children in Jamma district, in South Wollo zone, Amhara Region in Ethiopia. The district covers approximately 129,281.25 hectares. It is bordered on the South by North Shewa (Mida Oromo), on the West by Kolella district, on the north by Woreillu and on the east by North Shewa (Gera district). In 2018, the district had a total population of 148,152, of which 72,592 were women and 75,558 men. The total number of children aged 1–9 years was 29,338, of which 17,013 were males and 12,325 females [24].

Jamma is found at a distance of 260 km and 120 km from the cities of Addis Ababa and Dessie, respectively the annual rainfall is 1,130 mm. The administration of the district is divided into 22 rural and 2 urban *kebeles.* A *kebele* is the smallest local administrative unit in Ethiopia, with a population of roughly 5,000 people. In Jamma district, there is one governmental primary hospital, 6 public health centers and 7 private clinics. There is a health post in every *kebele*. Each health post is run by two female health extension workers.

### Study design, sample size and sampling procedures

In this community-based cross-sectional study, data was collected from January 1, 2019 to March 30, 2019. Sample size was determined using the assumptions of population-based prevalence surveys with the gold standard cluster random sampling methods as described elsewhere [25]. We calculated the sample size for cluster random sampling with estimated prevalence (*p*) of active trachoma 53.9% among children aged 1–9 years taken from a similar area in Ankober district, Amhara Region [26], a margin of error (*w*) of 5%, Z_α_/_2_ is 1.96 at 95% CI (confidence interval) and a design effect of 1.5 due to the multi-stage sampling we used. Then by adding a 10% non-response rate, the final sample size was 640.

Using the gold standard of trachoma prevalence study [25], a two-stage sampling method was used. In the first stage, six rural *kebeles* were selected randomly out of rural 22 *kebeles*. In our study, one *kebele* is considered as one cluster. Then, the modification of cluster random sampling method of probability proportional-to-size sampling based on the cluster population was used to determine the required sample size from each of the selected *kebeles*.

The sampling units were households that had at least one child 1–9 years old. Health extension workers in the selected *kebeles* provided a list of households that had at least one child of the proper age to be included in the sampling frame. Then, those households with children aged 1–9 years were coded to easily differentiate them during house-to-house visits at the time of data collection. The health extension workers were able to provide their trachoma antibiotic treatment logs; the antibiotic treatment log is regularly updated during the maternal follow-up for any vaccination of the child, so it unlikely to miss any households with children.

During the second stage, systematic sampling with a fixed interval of every 4^th^ house was used to select sampling unit households within each cluster. To select the house-paired with children aged 1–9 years, the data collector started with a bench mark of the known location (randomly selected household) and then walked straight forward (moving towards the front) to identify each house.

Although the current ‘gold standard’ of trachoma prevalence survey recommends all children and adults within the cluster be included [25], we did not apply this since our study target population was limited to children aged 1–9 years. Furthermore, when more than one child of the target age was present in a selected household, a lottery method was used to select the participant child to estimate the prevalence for the study area relative to the total sample size. When the child and/or mother/caregiver was not available from the selected household upon the first visit, another visit was made the same day. If they were again not available, a third visit was performed the next day in order to minimize the non-response rate. If not available after the three visits, the participant was taken as non-respondent.

### Outcome measurement

To be in line with current global guidance and to make the findings of this study useful for the national trachoma elimination program, TF and TI were studied separately, with TF being the primary outcome of interest for this study, since as the indicator on which decisions are made (start MDA [mass-drug-administration], stop MDA, elimination threshold) are all based on TF [1, 25].

To measure the outcome variable active trachoma, a clinical eye examination for the presence or absence of TF and TI was performed using methods from a simple system for the assessment of trachoma and its complications and the standard WHO simplified grading system [9, 27]. Therefore, the clinical eye examination took place at the time of the survey. Integrated eye care workers (IECWs) examined each eye separately by using binocular examination loupes lenses (× 2.5 magnifications) manufactured by Donegan Optical Company, Inc. USA. The examination of the eye was done by careful inspection of eye lashes, cornea, limbus, eversion of the upper lid and the tarsal conjunctiva. First, the right eye was examined followed by the left to avoid failure to recall in which eye the examiner saw an abnormality.

Based on the eye examination result, we classified a child’s trachoma as TF or TI. Child with TF meant the presence of 5 or more follicles in the central part of the upper tarsal conjunctiva, each at least 0.5 mm in diameter, whereas child with TI was the presence of pronounced inflammatory thickening of the upper tarsal conjunctiva that obscured more than half of the normal deep vessels [9, 27].

### Data collection procedures and quality assurance

The data collection tools were a pre-tested structured questionnaire, on-the-spot observational checklist and clinical examination for active trachoma. The questionnaire, which asked about socio-demographic and WASH (water, sanitation and hygiene) factors, was developed in English, translated into Amharic and then translated back into English by language experts to ensure consistency. Six Environmental Health Professionals who had BSc degree collected data using pre-tested structured questionnaire and on-the-spot observational checklist.

The questionnaire was pre-tested on 10% of the sample size (64 participants) in one rural area in Jamma district apart from the selected *kebeles*. The pre-test was conducted by the data collectors in order to improve their skills during the actual data collection. Any changes to the protocol were made on the basis of the pre-test. During the data collection period, the collected data were checked on a daily basis for completeness by the principal investigator and supervisors and any incomplete data were re-collected the same day. Double data entry was performed to minimize error during this step.

Three integrated eye care workers (IECWs) who were diploma nurses and certified by Ethiopia’s Ministry of Health in identifying active trachoma act as IECWs (also called graders) to diagnose and grade trachoma accurately. IECWs are also called graders who followed tropical data approach methods for identifying TF and TI. The study participant children in the family were examined for trachoma, and their mothers/caregivers (aged 18 years or older) responded to the household survey questionnaire through face-to-face interviews. The various data quality assurance methods were performed such as pre-test of the survey tool, training of data collector, daily supervision during data collection, double data entry, and data cleaning before analysis. The data quality assurance methods used for household survey in this study were similar to other studies as described elsewhere [28–30].

### Data management and analysis

Data were analysed using SPSS (Statistical Package for Social Sciences) Version 25.0. A logistic regression model with 95% CI were used. Descriptive statistics such as frequency distribution and prevalence were computed. The prevalence of TF and TI was estimated by dividing the number of TF and TI positive cases by the total study participants and then multiplying by 100. Data were analysed using binary logistic regression model to identify factors associated with TF.

During the analysis, independent variables having *p*-value < 0.25 from bivariable analysis (Crude odds ratio [COR] at 95% CI) were selected and retained into multivariable logistic regression analysis. A *p*-value < 0.25 was used as a cut-off-point for selection of potential confounders for candidates to be adjusted in multivariable analysis as described by Hosmer and Lemeshow [31]. From the adjusted multivariable analysis, variables with *p*-value < 0.05 with adjusted odds ratio (AOR) at 95% CI were declared as statistically significant and independent factors of TF. Adjustment for the selected cluster *kebeles* was not done since all the clustered areas were rural and found in one district, which has homogeneous characteristics.

Multi-collinearity between independent variables was checked using a standard error of the coefficient with cut-off-point 2.0 [31]; we found a maximum standard error of 1.9, which indicated no collinearity between independent variables. Hosmer and Lemeshow goodness-of-fit test [31] was used to check the model fitness of the adjusted multivariable analysis with cut-off-point *p*-value > 0.05 and found to be *p*-value 0.87.

## Result

### Socio demographic characteristics

The response rate of this study was 96.25%. Among 616 children who participated in the study, 313 (50.8%) were male. The age distribution of the study participant children was 269 (43.7%) from 12-36 months, 171 (27.8%) from 37-48 months and 176 (28.5%) > 48 months. The mean age of the children was 41.8 ± 19.8 months. The mean number of children 1–9 years old in a given household was 1.7 ± 0.7 children (Table 1).

**Table 1:** Socio-demographic characteristics and bivariable analysis with TF among children 1–9 years of age in rural Jamma District, northeastern Ethiopia, January-March, 2019

**Variable**	**Frequency**	**TF (** ***N *= 616)**		**COR (95% CI)**	***p-*** **value**
		**Yes**	**No**		
	***n*** ** (%)**	***n***	***n***		
**Child’s primary caregiver**					
Mother	488 (79.2)	55	433	1	
Father	60 (9.8)	6	54	0.87 (0.36 - 2.13)	0.760
Other	68 (11.0)	6	62	0.76 (0.32 - 1.84)	0.541
**Sex of household head**					
Female	93 (11.1)	11	82	1	
Male	523 (84.9)	56	467	0.89 (0.45 - 1.78)	0.742
**Religion of household head**					
Christian	318 (51.6)	38	280	1.25 (0.76 - 2.10)	0.371
Muslim	298 (48.4)	29	269	1	
**Age of mother/caregiver (years)**					
18-29	218 (35.4)	28	190	1	
30-44	351 (57.0)	31	320	0.65 (0.38 - 1.13)	0.121
≥ 45	47 (7.6)	8	39	1.39 (0.59 - 3.28)	0.453
**Mother/caregiver literacy**					
Illiterate^c^	483 (78.4)	59	424	2.17 (1.31 - 6.83)	0.042
Read and write	133 (21.6)	8	125	1	
**Occupation of mother/caregiver**					
Farming and rearing domestic animals^a^	443 (71.9)	49	394	0.99 (0.45 - 2.19)	0.990
Rearing domestic animals only	35 (5.7)	5	30	1.33 (0.40 - 4.42)	0.637
Civil servant or daily labourer	66 (10.7)	5	61	0.65 (0.20 - 2.12)	0.484
Merchant	72 (11.7)	8	64	1	
**Household size (persons)**					
3-5	196 (31.8)	6	190	1	
6-7	264 (42.9)	14	250	1.77 (0.67 - 4.70)	0.241
> 7	156 (25.3)	47	109	7.65 (4.65 - 12.98)	< 0.001
**Average monthly income** ^b^ ** (USD)**					
< $53.8	441 (71.6)	56	385	2.17 (0.98 - 3.85)	0.052
≥ $53.8	175 (28.4)	11	164	1	
**Age of child (months)**					
12-36	269 (43.7)	27	242	1.04 (0.55 - 1.98)	0.891
37-48	171 (27.7)	23	148	1.45 (0.75 - 2.83)	0.273
> 48	176 (28.6)	17	159	1	
**Child gender**					
Female	303 (49.2)	43	260	1.99 (1.18 - 3.37)	0.011
Male	313 (50.8)	24	289	1	
**Number of children age 1-9 years per household**					
One	475 (77.1)	13	462	0.04 (0.02 - 0.09)	< 0.001
Two or more	141 (22.9)	58	83	1	
**Birth order of participant child**					
First	389 (63.2)	37	352	1	
Second	172 (27.9)	21	151	1.32 (0.75 - 2.34)	0.330
Third or above	55 (8.9)	9	46	1.86 (0.84 - 4.10)	0.121
**Domestic animals kept overnight in the home** ^b^					
Yes	127 (20.6)	52	75	9.56 (11.74 - 18.8)	< 0.001
No	489 (79.4)	15	474	1	

1, reference category; COR (95% CI), crude odds ratio at 95% CI (confidence interval)

*TF* trachomatous inflammation-follicular

^a^Domestic animals included goat, sheep, chicken, cow, horse and donkey

^b^The average exchange rate of $1 USD (United States Dollars) was 27.87 ETB (Ethiopian birr) during January-March, 2019

^c^Illiterate means a study participant child’s mother/caregiver had not attended formal or informal education and was unable to read and write

Almost three-fourths (71.9%) of heads of households reported an occupation of farming and rearing domestic animals. Just over half 318 (51.6%) were Christians. Mothers/caregivers of 483 (78.4%) children illiterate. The mean family size was 5.5 ± 1.9 persons. The minimum and maximum family size was 3 and 13, respectively. About one-fifth (20.6%) of households kept domestic animals overnight in the same room as family (Table 1).

### Prevalence of active trachoma

Prevalence of TF was 10.9% (95% CI [8.6–13.6%]) among the rural children aged 1–9 years and the prevalence of TI was 2.1% (95% CI [0.95–3.25%]).

### Water, sanitation and hygiene (WASH) and bi-variable analysis with trachomatous inflammation-follicular (TF)

The common sources of water for domestic consumption were protected wells (*n* = 332, 53.9%), protected spring (*n* = 180, 29.2%) and unprotected spring (*n* = 104, 16.9%). About two-thirds (*n* = 370, 60.1%) of children were from households having water access within 30 min or less travel time on foot and a majority (*n* = 556, 90.3%) of children were from households that consumed less than 15 L water per person per day. Almost one-sixth (*n* = 108, 17.5%) of the children involved in this study had an ocular discharge (Table 2).

**Table 2:** Water and sanitation characteristics and bivariable analysis with TF among children 1–9 years of age in rural Jamma District, northeastern Ethiopia, January-March, 2019

**Variable**	**Frequency**	**TF (** ***N *= 616)**		**COR (95% CI)**	***p*** **-value**
		**Yes**	**No**		
	***n*** ** (%)**	***n***	***n***		
**Source of water**					
Protected well	332 (53.9)	10	322	0.07 (0.03 - 0.15)	0.001
Protected spring	180 (29.2)	25	155	0.36 (0.20 - 0.66)	< 0.001
Unprotected spring	104 (16.9)	32	72	1	
**Water consumption/person/day (litres)**					
< 15	556 (90.3)	62	494	1	
≥ 15	60 (9.7)	5	55	0.72 (0.28 - 1.88)	0.510
**Travel time on foot to obtain water (minutes)**					
≤ 30	370 (60.1)	15	355	0.15 (0.09 - 0.29)	0.001
> 30	246 (39.9)	52	194	1	
**Latrine access**					
Yes	527 (85.6)	26	501	0.06 (0.03 - 0.11)	< 0.001
No	89 (14.4)	44	45	1	
**Human feces observed in the compound**					
Yes	131 (21.3)	48	83	8.18 (3.94 - 16.34)	< 0.001
No	485 (78.7)	19	466	1	
**Child had ocular discharge**					
Yes	178 (28.9)	59	119	26.65 (14.23 - 44.35)	< 0.001
No	438 (71.1)	8	430	1	
**Child had nasal discharge**					
Yes	279 (45.3)	62	217	18.97 (9.23 - 27.03)	< 0.001
No	337 (54.7)	5	332	1	

1, reference category; COR (95% CI); crude odds ratio at 95% CI (confidence interval)

*TF* trachomatous inflammation-follicular

A majority 527 (85.6%) of the households had latrine access, whereas 89 (14.4%) had no latrine. We also found that in one-fifth (21.3%) of the households, human feces was observed in the compound, whereas no human feces was observed in 79.7% of households' compounds. Two-thirds of the children (*n* = 424, 68.8%) washed their hands once per day, while 192 (31.2%) washed them twice or more per day. Just over half 344 (55.8%) washed their faces once per day. A majority 361 (58.6%) of children did not use soap for washing of faces and hands (Table 3).

**Table 3:** Children’s hygiene-related factors and bivariable analysis with TF among children 1–9 years of age in rural Jamma District, northeastern Ethiopia, January-March, 2019

**Variable**	**Frequency**	**TF (** ***N *= 616)**		**COR (95%CI)**	***p*** **-value**
		**Yes**	**No**		
	***n *(%)**	***n***	***n***		
**Child frequency of hand washing per day**					
None or once	424 (68.8)	47	377	1.07 (0.62 - 1.87)	0.800
Twice or more	192 (31.2)	20	172	1	
**Child frequency of face washing per day**					
None or once	344 (55.8)	42	302	1.37 (0.82 - 2.32)	0.231
Twice or more	272 (44.2)	25	247	1	
**Child’s use of soap during hand and face washing**					
No	361 (58.6)	57	304	4.59 (2.29 - 9.18)	< 0.001
Yes	255 (41.4)	10	245	1	
**Frequency of child’s bathing/week**					
None or once	363 (58.9)	52	311	2.65 (0.81 - 4.83)	0.348
Twice or more	253 (41.1)	15	238	1	
**Frequency of child’s clothes being washed/week**					
None or once	337 (54.7)	45	292	1.80 (1.05 - 3.08)	0.030
Twice or more	279 (45.3)	22	257	1	

1, reference category; COR (95% CI); crude odds ratio at 95% CI (confidence interval)

*TF* trachomatous inflammation-follicular

### Factors significantly associated with trachomatous inflammation-follicular (TF) from multivariable analysis

After controlling for confounding factors from multivariable analysis, we found that factors significantly associated with active trachoma were family size greater than 7 persons per household, caregiver illiteracy, presence of domestic animals kept overnight within the home, presence of observable feces in the compound, and presence of ocular and nasal discharge found to be (*p* < 0.05) (Table 4).

**Table 4:** Factors significantly associated with TF among children 1–9 years of age from multivariable logistic regression analysis

**Variable**	**TF (*N *= 616)**		**AOR (95% CI)**	***p*** **-value**
	**Yes**	**No**		
	***n***	***n***		
**Mother/caregiver literacy**				
Illiterate	59	429	2.01 (1.11 - 4.67)	0.042
Read and write	8	125	1	
**Household size (persons)**				
3-5	6	190	1	
6-7	14	250	0.91 (0.15 - 5.45)	0.921
> 7	47	109	3.50 (1.66 - 8.50)	0.010
**Observable feces in the house compound**				
Yes	48	83	5.10 (2.01 - 10.14)	< 0.001
No	19	466	1	
**Domestic animals kept overnight in the same room as family**				
Yes	52	75	4.32 (2.49 - 9.52)	< 0.001
No	15	474	1	
**Soap use during washing of child’s hands and face**				
No	57	304	2.41 (1.29 - 5.18)	0.003
Yes	10	245	1	
**Child had ocular discharge**				
Yes	59	119	7.23 (4.10 - 12.51)	< 0.001
No	8	430	1	
**Child had nasal discharge**				
Yes	62	217	4.54 (1.95 - 9.26)	< 0.001
No	5	332	1	

1, reference category; AOR (95% CI); adjusted odds ratio at 95% CI (confidence interval)

*TF* trachomatous inflammation-follicular

Of socio-demographic factors, the odds of developing active trachoma among children who had an illiterate caregiver were 2.01 times (AOR = 2.01; 95% CI [1.11–4.67]) higher than among children whose caregiver could read and write. The odds of having TF in children who were from a household size > 7 were 3.50 times (AOR = 3.50, 95% CI [1.66–8.50]) higher than in children from a household size of 3–5. The odds of having active trachoma among children from a household in a compound containing observable feces were 5.10 times higher than for their counterparts without feces observable in the compound (AOR = 5.10, 95% CI [2.01–10.14]) (Table 4).

Children whose parents’ kept domestic animals overnight in the same room as the family were 4.32 times more likely to develop TF than those whose domestic animals were kept overnight in a separate room (AOR = 4.32, 95% CI [2.49–9.52]). Children who had ocular discharge were 7.23 times (AOR = 7.23, 95% CI [4.10- 12.51]) more likely to develop TF than those children who had no ocular discharge. Children who had nasal discharge were 4.54 times (AOR = 4.54, 95% CI [1.95–9.26]) more likely to develop TF than those children who had no nasal discharge. Children whose hands and face were washed without soap also were also 2.4 times (AOR = 2.41; 95% CI 1.29–5.18) more likely to have TF than those children whose hands and face were washed with soap (Table 4).

## Discussion

This community-based cross-sectional study was designed to assess the prevalence of TF and associated factors among children aged 1–9 years in rural communities of Jamma district in Ethiopia. We found that the prevalence of TF was 10.9% among the rural children aged 1–9 years. Family size of above 7 persons, household literacy (illiterate), presence of domestic animals kept overnight in the same room as the family and presence of observable feces in the house compound were factors significantly associated with TF.

The prevalence of TF in this study is quite similar to that found in studies done in Leku Town, Southern Ethiopia (11.1%) [32] but lower than that found in the studies conducted in Woliso Town (20.4%) [21], Madda Walabu Town (22.0%) [33], Mojo and Lume district (22.8%) [22] and Gonji Kolella district (23.1%) [16]. This difference might be due to the fact that our findings were made after more than a decade of combined efforts of Ethiopia’s governmental and non-governmental organizations (NGOs) to reduce the prevalence of trachoma by increasing coverage of mass antibiotic distribution, improving integration of health promotion with primary eye care, and providing health information within local districts on personal and environmental hygiene.

Our study also shows that the prevalence of TF is lower than found by the study conducted in Baso Liben District of East Gojjam, 24.1% [20], Yobe state of Nigeria, 35.7% [34] and Gazegibela district, Amhara Region, 52.4% [35]. The higher prevalence in these other areas might be due to due to poorer hygiene practices, differences in infrastructure and health service coverage, endemicity of the trachoma, period of study, and geographical and cultural factors. In the Gazegibela district, there might also be other related factors, including the fact that it is repeatedly affected by drought and is one of the food insecure districts of the Amhara region. A study about trachoma elimination approaches, experiences and performance of interventions in Amhara Regional State, Ethiopia also showed interventions are effective in reducing trachoma [36]. Hence, the current reduction of trachoma prevalence in our study might be due to the implementation of the rural and urban health extension program [37], the Ministry of Health Trachoma Control and Elimination Program [36], national ONE WASH program [38, 39], the Carter Center Trachoma Control and Elimination Program in Ethiopia since 1997 [40] and implementation of the World Health Organization (WHO) endorsed SAFE strategy for trachoma control [41].

The current study shows that living in a household with a family size greater than seven was associated with TF among children aged 1–9 years. This is consistent with a study done in Gonji Kolella district of West Gojjam zone [16]. Furthermore, our findings are in agreement with a study conducted in Ankober, Ethiopia [26] and Leku Town Southern Ethiopia [32]. This might be due a larger family size creating crowded living conditions, which may compromise cleanliness of the sanitation facilities and create a shortage of water for washing children’s hand and faces. Water supply interruptions also may reduce the hygienic status of children within at larger family [28, 42]. When there is larger family, affording soap may also be difficult due to economic constraints [43], which in turn hinders the use of soap when washing hands and face [44].

We also found that having an illiterate mother/caregiver was associated with TF among children aged 1–9 years. The reason might be that an educated mother/caregiver might be more aware of the benefits of hygiene practices and health services to their children compared to an uneducated mother. Also, an uneducated mother/caregiver may not keep sanitary facilities clean [28]. Being un-educated also may hinder the effectiveness of the implementation of the SAFE strategy to prevent trachoma [41]. The active involvement of health professionals in hygiene and sanitation is crucial to accelerating and consolidating progress in disease prevention [45]; it might be found that changing the behaviour of mothers/caregivers who have been educated in how to prevent trachoma is easy.

In this study, keeping animals overnight in the same room as the family was significantly related to the prevalence of TF among children aged 1–9 years. This may be related to animal dung being a breeding site for flies to which nearby that cause an increase in the exposure of children are then increasingly exposed. The flies are attracted to eyes that are red and have discharge, and then carry the bacteria *Chlamydia trachomatis* to the eyes of others within the family or the community, both children and adults. The study also revealed that the presence of observable feces in the living compound was significantly associated with TF. This finding is in line with that of a study done in north-eastern Nigeria [34]. This is apparently due to isolated human feces on the soil surface being the best larval medium for Musca sorbens, the vector for trachoma.

The absence of ocular and nasal discharge was inversely related to the presence of TF. It can be argued that the absence of ocular and nasal discharge decreases the acquisition and transmission of trachoma since they are related to decreased frequency of fly-eye contacts by the trachoma vector Musca sorbens. Studies conducted at Vietnam [46], Woliso Town [21] and South Wollo in Ethiopia [13] revealed findings similar to our study.

In contrast to several other studies conducted in Ethiopia, our study findings strengthen evidence of the problem in Ethiopia of domestic animals being kept overnight in the same room as the family as a main factor of TF; this is a good input for further study (presumably with interventional randomized trials) before advocating any wider public health intervention. In rural Ethiopia, including rural Jamma district, the livelihood of farmers is the production of domestic animals (cattle, goats, sheep, chickens, horses and donkeys) and therefore interventions that support having a clean and partitioned building in which to keep domestic animals overnight as a method to tackle trachoma might be a good insight.

### Limitations of the study and gaps for further studies

This study had some limitations. The study may be prone to social desirability bias because some of the data on independent variables was obtained by self-reporting. A study in Ethiopia showed studies in Ethiopia faced underreporting due to social desirability bias [47]. Therefore, an observational follow-up study triangulated with qualitative data is recommended to minimize social desirability bias on the part of study participants and to provide more evidence on factors associated with TF. Six clusters were included in this study and the authors realized later that this number was insufficient to estimate prevalence accurately using a conventional statistical approach. We sampled just one child per household when there could have been more children in the same household. This might have introduced bias during selection of child with or without TF during data collection. Further study is highly recommended to include all children in the households rather than selecting just one.

The other limitation was that eye discharge may be a *proxy* factor for the occurrence of active trachoma and the reverse causality bias may be a probable explanation for ocular and nasal discharge associated with TF. Therefore, further studies should focus on investigating distal, intermediate and proximal factors to effectively prevent active trachoma. We also did not study household, individual and community factors using multilevel analysis, all of which will help to design appropriate interventions; thus, it is recommended that researchers address this limitation. Exploring the pathway between having domestic animals and children getting TF is also highly recommended.

Furthermore, the relationship between seasonal variation and trachoma was not addressed; it is recommended that researchers address this factor since WASH conditions depend on seasonal variation [48]. For instance, during the dry season, there may be an intermittent water supply [42], which will hinder washing of hands and faces. This study's target population was children aged 1–9 years only, which is not in line with the current WHO gold standard survey method for trachoma; it recommends examination of all children 1–9 years and all adults ages 15 and above [1, 25]. Therefore, further studies are highly encouraged per the gold standard for trachoma study.

## Conclusion

The prevalence of TF among children aged 1–9 in rural communities was found to be twice as high as the WHO-recommended trachoma elimination goal of less than 5%. Our findings showed domestic animals kept overnight in the same room as family, mother/caregiver illiteracy, household size of greater than 7 persons, and presence of observable feces in the house compound were factors significantly associated with TF among children aged 1–9 years in rural Jamma District in Ethiopia.

Despite SAFE interventions implemented for over a decade, our findings show the prevalence of TF is still above the WHO elimination targets, likely due to very high baseline prevalence and related to the identified factors. It will require major investments and political will to address these factors throughout trachoma-endemic rural Ethiopia, particularly in northeastern Ethiopia. To meet the WHO targets, we recommended interventions be made that strengthen all aspects of the integrated strategy endorsed by WHO (SAFE), such as surgery for trichiasis, antibiotic therapy, facial cleanliness and, most importantly, environmental improvements. 

Although the cultural/emotional attachment of families to their domestic animals is a challenge to overcome, we recommend domestic animals be kept overnight in a separate room to prevent TF among children aged 1–9 years in rural Jamma district, Ethiopia. We also recommend that policy makers design mechanisms to enhance overall living conditions in rural Ethiopian communities, with a priority on encouraging behavioural change among householders about keeping household compounds clean and creating awareness among mothers/caregivers about ways to prevent trachoma.

### Supplementary Information

**Additional file 1.** English version of the questioner.

## Data Availability

The datasets collected and/or analyzed during the current study are available from the corresponding author on reasonable request.

## Abbreviations

AOR: Adjusted odds ratio
COR: Crude odds ratio
CSA: Central statistical agency
TF: Trachomatous inflammation-follicular
TI: Trachomatous inflammation-intense
SAFE: Surgery, antibiotics, facial hygiene and environmental improvement
IECWs: Integrated eye care workers
WHO: World Health Organization
