# Supplementary material for: Prevalence of trachomatous inflammation-follicular and associated factors among children aged 1-9 years in northeastern Ethiopia
Source: BMC Pediatr. 2024 Feb 19;24:128. doi: 10.1186/s12887-024-04587-4 (PMC10875859; doi:10.1186/s12887-024-04587-4)
Supplement: Supplementary file 1 — Additional file 1. English version of the questioner. [file 12887_2024_4587_MOESM1_ESM.docx]

# English version of the questioner

House code: _________kebele:___________ Gott: _____

Name of observer/examiner/interviewer: _________________

Signature: ___________ Date: ____________

Name of supervisor: _________________ Signature: _________ Date: ____________

Q. ID _________

**Socio-demographic characteristics of respondents (100)**

| Caregiver related | | | |
| --- | --- | --- | --- |
| code | Questions | Possible responses | comment |
| 101 | Who is the primary caregiver of children in this household? | 1 = Mother  2 = Father  9 = Other/specify………………. |  |
| 102 | Age (year)? | ……………….. |  |
| 103 | Sex of head of the house? | 1 = Female  2 = Male |  |
| 104 | Marital status? | 1 = Single  2 = Married  3 = Divorced  4 = Widowed |  |
| 105 | Religion? | 1 = Orthodox Christian  2 = Muslim  3 = Catholic  4 = Protestant  9 = Other/specify……………………………………. |  |
| 106 | Household average Monthly income? | ……………… |  |
| 107 | Educational status of care giver? | ……………… |  |
| 108 | Household size? | ………………….. |  |
| 109 | Occupation? | 1 = Farming and cattle raring  2 = cattle rearing only  3= Civil servant  4 = Merchant  5 = Daily laborer  9 = Other/specify………………………………….. |  |
| 110 | For how long you stayed in these got? | 1 = below one year  2 = 1-4 years  3 = above 4 years |  |
| Children 1-9 years related | | | |
| 111 | Sex of child | 1. Female 2. Male |  |
| 112 | Age of the child (months) | ………………………… |  |
| 113 | Number 1-9 years age children in the household? | ……………………… |  |
| 114 | Birth order of the child (rank)? | ………………………… |  |

**Water, sanitation and hygiene practices (200)**

| S.n | Questions | Possible responses |  |
| --- | --- | --- | --- |
| 201 | What is your primary source of water for different purposes? | 1 = protected well  2 = protected spring  3 = unprotected spring  9 = Other/ specify………………………….. |  |
| 202 | How many liters of water used per day per person? | …………L |  |
| 203 | Time taken from home to water sources | ……………….min. |  |
| 204 | Are you keeping domestic animals with you in the same house? | 0 = No  1 = Yes |  |
| 205 | Do you think personal hygiene and sanitation are important to prevent trachoma? | 0 = No  1 = Yes |  |
| 206 | Do you have a latrine? | 0 = No  1 = Yes |  |
| 207 | If no to Q-211 where does the family defecate? | 1 = Open field  9 = Other /specify…………………………………… |  |
| 208 | Ownership of the latrine? | 1 = Private  2 = shared |  |
| 209 | Is the latrine functional? (observation) | 0 = No  1 = Yes |  |
| 210 | Is there any hand washing container near the latrine? (observation) | 0 = No  1 = Yes |  |
| 211 | How close the hand washing container is to the latrine? /Observation) | 1 = Next to the latrine  2 = Within walking distance  3 = Inside the house  9 = other/specify |  |
| 212 | Are there observable faeces in the compound? | 0 = no  1 = yes |  |
| 213 | Distance of latrine from home? | ……….m. |  |
| 214 | Where do you dispose of the feces of children who have not started using latrines? | 1 = in the house  2 = In the compound  3 = Outside the compound  4 = In the latrine  9 = Other, specify |  |

Behavioral factors (300)

| S.n | Questions | Possible responses | Comments |
| --- | --- | --- | --- |
| 301 | Do you use Soap to wash your child’s face and hands? | 0 = no  1 = yes |  |
| 302 | How many times you wash your children’s hands per day? | ………… |  |
| 303 | How often do you wash your children’s faces per day? | ………… |  |
| 304 | How often do you wash your children’s clots for the last one month? | ………… |  |
| 305 | How often do you bath your children for the last one month? | …………. |  |

Trachoma clinical assessment form (outcome measurement) (400)

| No | Eye examination findings among 1-9 year - old children | | | |
| --- | --- | --- | --- | --- |
|  | Ocular discharge | Nasal discharge | TF | TI |
| 401 | 0 = No  1 = yes | 0 = No  1 = yes | 0 = No  1 = yes | 0 = No  1 = yes |

Trachoma status (500)

| 501 | Trachoma status | 0 = No  1 = yes |  |
| --- | --- | --- | --- |

Thank you very much for your participation in this study
